# The ontology of genetic susceptibility factors (OGSF) and its application in modeling genetic susceptibility to vaccine adverse events

**DOI:** 10.1186/2041-1480-5-19

**Published:** 2014-04-30

**Authors:** Yu Lin, Yongqun He

**Affiliations:** 1 Unit for Laboratory Animal Medicine, University of Michigan Medical School, Ann Arbor, MI 48109, USA; 2 Department of Microbiology and Immunology, University of Michigan Medical School, Ann Arbor, MI 48109, USA; 3 Center for Computational Medicine and Bioinformatics, University of Michigan Medical School, Ann Arbor, MI 48109, USA

## Abstract

**Background:**

Due to human variations in genetic susceptibility, vaccination often triggers adverse events in a small population of vaccinees. Based on our previous work on ontological modeling of genetic susceptibility to disease, we developed an Ontology of Genetic Susceptibility Factors (OGSF), a biomedical ontology in the domain of genetic susceptibility and genetic susceptibility factors. The OGSF framework was then applied in the area of vaccine adverse events (VAEs).

**Results:**

OGSF aligns with the Basic Formal Ontology (BFO). OGSF defines ‘genetic susceptibility’ as a subclass of BFO:disposition and has a material basis ‘genetic susceptibility factor’. The ‘genetic susceptibility to pathological bodily process’ is a subclasses of ‘genetic susceptibility’. A VAE is a type of pathological bodily process. OGSF represents different types of genetic susceptibility factors including various susceptibility alleles (*e.g.*, SNP and gene). A general OGSF design pattern was developed to represent genetic susceptibility to VAE and associated genetic susceptibility factors using experimental results in genetic association studies. To test and validate the design pattern, two case studies were populated in OGSF. In the first case study, human gene allele DBR*15:01 is susceptible to influenza vaccine Pandemrix-induced Multiple Sclerosis. The second case study reports genetic susceptibility polymorphisms associated with systemic smallpox VAEs. After the data of the Case Study 2 were represented using OGSF-based axioms, SPARQL was successfully developed to retrieve the susceptibility factors stored in the populated OGSF. A network of data from the Case Study 2 was constructed by using ontology terms and individuals as nodes and ontology relations as edges. Different social network analys is (SNA) methods were then applied to verify core OGSF terms. Interestingly, a SNA hub analysis verified all susceptibility alleles of SNPs and a SNA closeness analysis verified the susceptibility genes in Case Study 2. These results validated the proper OGSF structure identified different ontology aspects with SNA methods.

**Conclusions:**

OGSF provides a verified and robust framework for representing various genetic susceptibility types and genetic susceptibility factors annotated from experimental VAE genetic association studies. The RDF/OWL formulated ontology data can be queried using SPARQL and analyzed using centrality-based network analysis methods.

## Background

Genetic susceptibility, also called genetic predisposition, is an increased likelihood or chance of developing a particular disease (*e.g.*, diabetes) or pathological bodily process (*e.g.*, infection) due to the presence of one or more gene mutations with or without a family history of an increased risk [1]. Genetic susceptibility is associated with all kinds of disease and traits across the whole medical domain, such as infectious diseases [2], alcoholism [3], cancer [4], and autoimmunity [5]. As a more specific example, human vaccination may induce undesired adverse events, so called vaccine adverse event (VAE), which may be manifested in various forms of signs, symptoms and diseases [6]. The VAE may appear in a small population but not in the majority of vaccinee population, indicating the genetic susceptibility in the small population [7,8]. For example, compared to white children, the native American Indian Apache children have significant impairment of their antibody response to *H. influenzae* type b polysaccharide, thus they may be prone to develop adverse events if administered a *H. influenzae* vaccine with *H. influenzae* type b polysaccharide as its component [9]. Better understanding of genetic susceptibility factors to specific diseases will allow us design preventative and therapeutic measures to prevent and control the diseases in susceptible populations.

Various kinds of genetic variations bear susceptibilities, *e.g.*, linkage disequilibrium (LD, non-random association between two or more loci) haplotype, a linkage region, genetic polymorphisms, alleles and so on. These various genetic variant entities are named ‘genetic susceptibility factors’ by the authors. The allele that confers increased susceptibility may be inherited; whereas the disease itself will not. The single locus genotype is usually insufficient to cause a disease. A disease often appears when impaired expressions of alleles at other gene loci and/or environmental factors co-exist [10]. Genetic susceptibility factors might not have obvious mutations. A genetically inherited disorder is more likely the consequence of a polygenic combination of variants at several genes that might be common in healthy humans. Moreover, the main determinants of susceptibility may be different in different populations [11]. Furthermore, many environmental factors may interact with genetic factors, and they contribute to a diseased outcome simultaneously [7,12]. Many apparently contradictory findings in disease-gene association studies associated with different study designs increase the complexity of the problem [13]. The sophisticated nature of genetic susceptibility makes it challenging to identify true genetic factors associated with human susceptibility to a specific disease or a pathological bodily process.

The general methodology to identify the genetic susceptibility to complex disease is a combination of linkage and association studies in biological experimental science. At first, the family-based studies identify a linkage region contains several mega bases of DNA. To narrow down such a region to a susceptible gene (or genes), population-based case–control studies identify variants in linkage disequilibrium with the susceptibility locus, which lead to define the genomic region responsible for the original linkage signal [14]. Although the original linkage signal may not be detectable in some studies, combination of different levels of evidence from multiple studies may decipher true genetic susceptibility. At the post-genomics era, it is possible to use high throughput Omics methods to identify possible genetic variations that contribute to the genetic susceptibility. The strategy of applying Omics and other methods to study host genetic variations and their effects in vaccine-induced host immune responses (*e.g.*, VAEs) has been termed vaccinomics [12]. The notion of genetic susceptibility can be traced back to 1926 [15]. Since then, numerous literature reports of genetic susceptibility have been published. As of December 23, 2013, a PubMed search for “genetic susceptibility” has generated over 119,900 hits. However, a database of general genetic susceptibility factors is not yet available. As a first step towards systematically collecting and studying genetic susceptibility factors, there is a need to generate a consensus-based robust ontological framework for systematically representing and studying such genetic susceptibility and the genetic factors contributing to the susceptibility.

A formal ontology is a set of computer- and human- interpretable terms and relations that represent entities in a specific domain and how these entities relate to each other. Ontological terms are expressed in formal logic to support automated reasoning. Lin *et al.* have previously developed an Ontology of Genetic Susceptibility Factors to Diabetes Mellitus (OGSF-DM) intended to provide a framework for genetic susceptibility to diseases [14]. By using the *TCF7L2* gene and its susceptibility to Type 2 Diabetes (T2D) as an example, OGSF-DM formalizes the basic definitions of ‘genetic susceptibility’ and ‘genetic susceptibility factor’. The ontology OGSF-DM is a virtual ontology composed of three ontologies: the Ontology of Genetic Disease Investigation (OGDI), which imports other two ontologies: the Ontology of Glucose Metabolism Disorders (OGMD) and the Ontology of Geographical Regions (OGR). The previous study found out that essential variables impacting genetic susceptibility to diseases include: genetic polymorphism, the population and geographical location, the disease entities, and related statistical values (*e.g.*, odds ratio and p-value) [14].

The Open Biological and Biomedical Ontologies (OBO) Foundry community [16] has recently developed many ontologies that overlap the scope of OGSF-DM. For example, within the OBO Foundry, the Ontology for Biomedical Investigations (OBI) that represents biological and clinical investigations [17] overlaps with the scope of OGDI; the ontology Gazetteer (GAZ) that describes environmental places [18] overlaps with OGR. However, the ontological modeling of genetic susceptibility remains untouched. The original OGSF-DM was loosely aligned with BFO 1.0 by denoting some classes as subclasses of ‘continuant’ or ‘occurrent’. The structure of the three OGSF-DM ontologies did not follow the OBO Foundry’s principles [16], which makes it difficult to be integrated with other OBO Foundry ontologies. To leverage the reusability and interoperability of the community developed ontologies, we have found that the OGSF-DM would be better if being refined and focused more on the area of genetic susceptibility. We have thus proposed to develop a single ontology: the Ontology of Genetic Susceptibility Factors (OGSF), to represent various types of genetic susceptibility and genetic susceptibility factors supported by textual conclusions given by genetic association studies.

While the OGSF-DM modeled the genetic susceptibility to a disease (*i.e.*, diabetes mellitus) [14], genetic susceptibility is not always associated with only disease. In BFO, a disease is a subclass of disposition, which is positioned in the branch of BFO:continuant. The genetic susceptibility is often associated with the risk of a pathological bodily process including a vaccine adverse event [19-21]. The pathological bodily process as defined by the Ontology of General Medical Science (OGMS) as a process positioned under the branch of BFO:occurrent [22]. Therefore, the disease (a dependent continuant) and the pathological bodily process (a BFO:occurrent) are located in two different major branches of BFO. To more comprehensively represent entities related to genetic susceptibility, it is required for OGSF to represent pathological bodily processes such as vaccine adverse events.

In this paper, we introduce our development of a new version of genetic susceptibility-focused ontology: the Ontology of Genetic Susceptibility Factors (OGSF) by using BFO 2.0 as its upper ontology. To illustrate the ontology and verify our ontology design patterns, two vaccine adverse event-related genetic susceptibility case studies were specifically analyzed. Our studies demonstrate that the OGSF successfully provides an ontological framework for systematically representing genetic susceptibility, genetic susceptibility factors, associated entities and relations.

## Results

In what follows, single quotes are used to refer to a specific term within OGSF where appropriate. The numerical ID following the prefix of ontology is given after the term is mentioned, which gives the indication of the term’s resource. *Italics* are used to indicate the axioms or properties defined in the ontology.

### The new OGSF is aligned with BFO

**Figure 1:**
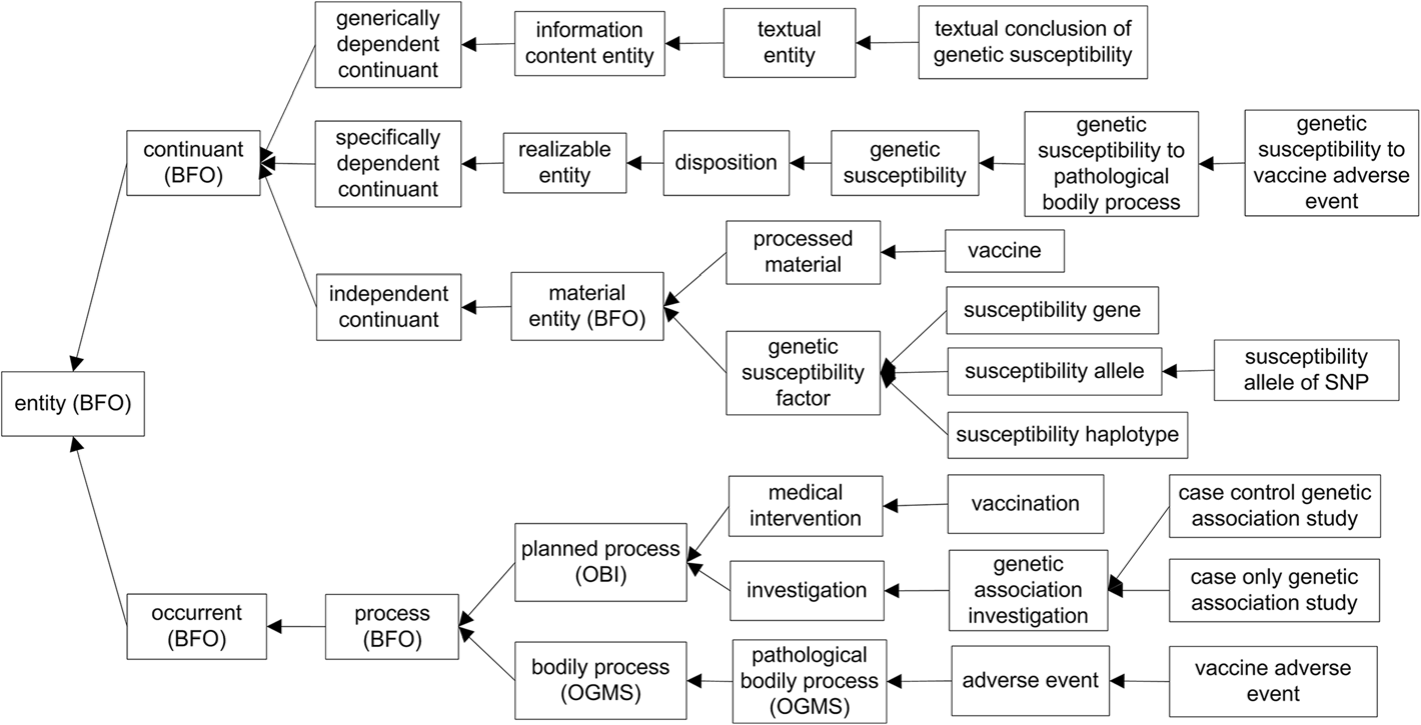
The OGSF hierarchy and key OGSF terms introduced in the paper.

The development of OGSF follows the OBO Foundry principles, including openness, collaboration, and use of a common shared syntax [16]. To align OGSF with BFO 2.0 version, we started with previously identified key terms and render them using BFO's terms as parent terms (Figure 1). To enable the reusability of other ontologies, we have imported many related terms and relations from existing OBO foundry ontologies. For example, the terms ‘vaccine’ (VO_0000001) and ‘vaccination’ (VO_0000002) are adopted from the Vaccine Ontology (VO) [23,24]; the terms ‘adverse event’ (OAE_0000001) and ‘vaccine adverse event’ (OAE_0000004) are imported from OAE. The relations between these vaccine terms and VAE terms are defined in the newly generated OVAE [8]. The vaccine related investigation is within the scope of the OBI, so that some OBI terms, such as ‘investigation’ and ‘textual conclusion’ were imported into OGSF.

In addition to the reuse of existing ontology terms, over 60 OGSF-specific class and property terms exist. The two OGSF core terms are: ‘genetic susceptibility’ and ‘genetic susceptibility factor’. The OGSF term ‘genetic susceptibility’ (OGSF_0000000) is a subclass of ‘BFO:disposition’ (BFO_0000016). The alternative term for ‘genetic susceptibility’ is ‘genetic predisposition’. In BFO 2.0, the genetic and other risk factors for specific diseases are considered as predispositions, *i.e.*, they are dispositions to acquire other dispositions. The realization of such a predisposition consists in processes which change the physical makeup of its bearer in such a way that parts of this bearer serve as the material basis for a disease [25]. Since the term ‘predisposition’ is not included in current version of BFO 2.0, we assert OGSF ‘genetic susceptibility’ as an immediate child of BFO term ‘disposition’. The child terms of ‘genetic susceptibility’ include: ‘genetic predisposition to disease of type X’ (OGMS_0000033) and ‘genetic susceptibility to pathological bodily process’ (OGSF_0000001). The term that reflects our use cases is ‘genetic susceptibility to vaccine adverse event’ (OGSF_0000010), which is a child term of ‘genetic susceptibility to pathological bodily process’.

Another OGSF core term ‘genetic susceptibility factor’ (OGSF_0000004) is a subclass of ‘material entity’ (BFO_0000040). Any allele, gene, genotype, or haplotype may be a genetic susceptibility factor if a genetic association study supports the association between any of those entities and a phenotype. The relation ‘material basis of at some time’ (BFO_0000127) is formalized in BFO 2.0 to represent the relation between a material entity and a disposition [25]. BFO 2.0 refers disposition to the potentials or powers of things in the world. Whenever a disposition exists, it is a disposition of something, namely its material bearer [25]. This relation is adopted to represent the relation between genetic susceptibility factor and genetic susceptibility in OGSF. At the instance level, the same genetic susceptibility factor bearing genetic susceptibility in a person has its entire existence all the time. But this statement may not be true at the class level. The same genetic susceptibility factor may contribute differently to the manifestation of a disease at different time periods and conditions. Such a meaning is reflected in the words ‘at some time’ of the relation ‘material basis of at some time’.

OGSF represents different types of genetic susceptibility factors, including haplotypes, genes, single nucleotide polymorphisms (SNPs), and alleles. A haplotype is a combination of DNA sequences at adjacent locations (loci) on a chromosome that can be inherited together from a single parent. A haplotype can describe a pair of genes on one chromosome or all genes on a chromosome from a parent. A haplotype can also refer to an inherited cluster of SNPs that are variations at single positions in the DNA sequence among individuals. An allele is an alternative form of the same gene or other genetic material that occupies a specific location on a chromosome. The Ontology for Genetic Interval (OGI) [26] defines different subclasses of allele including ‘allele of gene’, ‘allele of SNP’ and ‘allele of haplotype’. Since every individual has two parents who each contribute one allele, genetic susceptibility factors can usually be represented by the notion of allele. Sometimes two or more SNPs work together and contribute to genetic susceptibility. Two situations existed for this condition: the collaborative SNPs from one haplotype, and the synergistic combinations of SNPs from different haplotypes. Such cases are represented as ‘aggregate SNPs’ in OGSF. OGSF fully imports OGI, thus it inherits the OGI’s allele classes and definitions. OGSF inherits the OGI classification of haplotype, genes and SNPs as material entities containing sequence information [27]. Different from OGI, the DNA sequences in the Sequence Ontology (SO) represents sequence information itself [28]. The SO also does not differentiate different allele types. These are the reason why we use OGI instead of SO in OGSF. A new relation ‘is_allele_of_gene’ has been created to link ‘allele of gene’ and ‘gene’. This relation is required for logical definition and correct reasoning in susceptibility allele of gene analysis as shown in our Case Study 2 described later in the paper.

In total, OGSF contains over 600 class and property ontology terms as shown on http://www.ontobee.org/ontostat.php?ontology=OGSF. In our VAE susceptibility use case studies, we have also generated many OGSF instances as introduced later in this paper.

### Modeling genetic susceptibility to vaccine adverse event

As defined in the Vaccine Adverse Event Reporting System (VAERS) and Ontology for Adverse Event (OAE), a vaccine adverse event is an adverse event following vaccination and does not necessarily assume a causal association [8,20,21]. However, a causal association between administration of a specific vaccine and an adverse event in a particular population can be identified through systematic and statistical studies [7,12,29,30]. Although a large number of studies have provided supporting evidences for asserting susceptibility factors (*e.g.*, susceptibility alleles) to vaccine adverse event outcomes, the results of these studies cannot be automatically processed by computers. Our OGSF presentation aims to create a machine-interpretable ontological representation of these studies in order to analyze the results across studies and search for possible causal associations.

**Figure 2:**
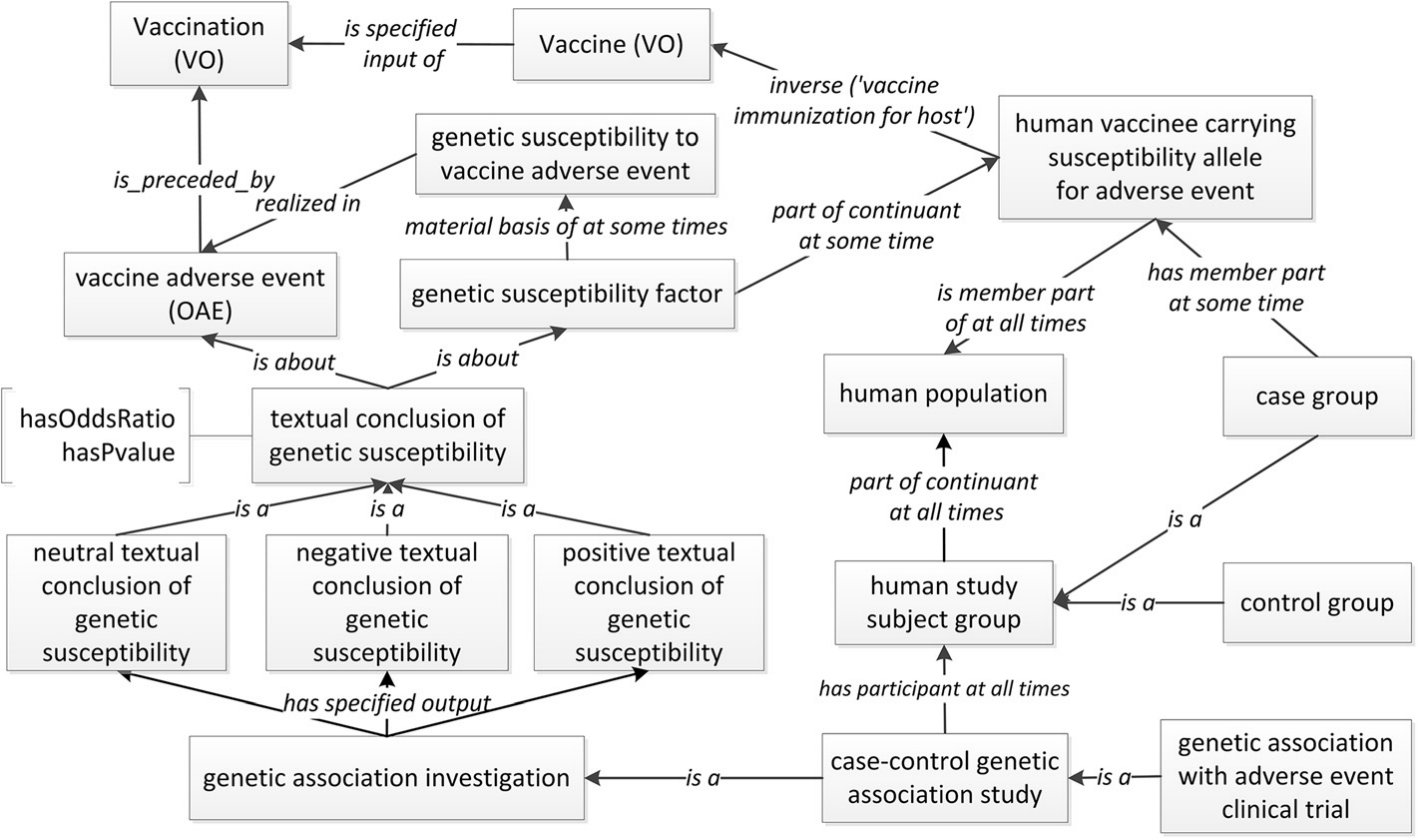
**General design of OGSF representing genetic susceptibility to vaccine adverse event.** Square boxes denote classes, and italicized terms along the arrows denote relations.

Figure 2 illustrates the design pattern of how OGSF is used to represent the association between a genetic susceptibility factor and a vaccine adverse event (VAE) based on experimental studies reported in the literature. As shown in the figure, the ‘genetic susceptibility factor’ is the material basis of ‘genetic susceptibility’. The ‘genetic susceptibility to vaccine adverse event’ is realized in the process of ‘vaccine adverse event’ (OAE_0000004). In the vaccine case, the genetic susceptibility factor is a part of a ‘human vaccinee carrying susceptibility allele for adverse event’ (OGSF_0000029), which ‘actively participates in’ the ‘vaccine adverse event’. As a participant of a ‘genetic association investigation’ (OGSF_0000016), a ‘case group’ (OGSF_0000022) has a member of ‘human vaccinee carrying susceptibility allele for adverse event’. A human vaccinee is vaccinated with a vaccine. The vaccination occurs before (or is preceded by) a vaccine adverse event. As a specified output of the genetic association investigation, the ‘textual conclusion of genetic susceptibility’ concludes the association between a ‘genetic susceptibility factor’ and a ‘vaccine adverse event’. Below we provide more specific details to introduce this OGSF design pattern.

The direct linkage from susceptibility-related terms to VAE terms is often required in our OGSF modeling. For example, in OGSF, we need to link ‘human vaccinee carrying susceptibility allele for adverse event’ (OGSF_0000029) to a vaccine. An object property term (ontological relation) reflecting such linkage is not available in existing ontologies. However, VO defines a shortcut relation ‘vaccine immunization for host’, which relates a vaccine with a vaccinee [23]. The strategy of designing and using shortcut relations has been promoted by Mungall *et al.* to simplify the complex axioms involving nested class expressions to make it ‘triple-friendly’ for complex OWL ontologies [31]. In our design, the ‘inverse’ usage of this VO shortcut relation ‘vaccine immunization for host’ connects from ‘human vaccinee carrying susceptibility allele for adverse event’ to ‘vaccine’.

The term ‘genetic association investigation’ (OGSF_0000016) is defined as: an investigation that aims to test whether single-locus alleles or genotype frequencies (or more generally, multi-locus haplotype frequencies) differ between two groups of individuals (usually diseased subjects and healthy controls). Different types of those studies exist. For example, a ‘case control genetic association study’ (OGSF_0000017) is a genetic association study that contains two types of human study subject groups: ‘case group’ and ‘control group’. The control group provides a background control in order to properly assess the results identified from the case group study. In contrast, a ‘case-only genetic association study’ (OGSF_0000036) includes a case group and does not have a control group to compare. The results obtained from a case-only genetic association study provide sufficient evidence to detect an association [32]. However, they are often biased by pre-condition of non-independence between the genetic and environmental factors in the population [33]. Another type of genetic association study is ‘family-based genetic study’ (OGSF_0000041) that investigates family members who may show different phenotypes. By analyzing entire genomes of people with a disease (cases) and similar people without the disease (controls), a Genome-Wide Association Study (GWAS or GWA study) examines many common genetic variants in different individuals to see the association between variant and a trait [7,12]. Such a genome wide association study is a type of ‘case control genetic association study’.

A reported genetic susceptibility study typically includes a conclusion of the association between a genetic factor and a disease (or pathologic bodily process) under specific conditions. Such conclusion is required to be represented ontologically. To represent the results from individual genetic association studies as reported in different papers, we have added an OGSF term ‘textual conclusion of genetic susceptibility’ to represent the textual conclusion of a genetic susceptibility study. Ontologically, a ‘textual conclusion of genetic susceptibility’ is asserted as a ‘specified output of’ a ‘genetic association investigation’. There are three types of ‘textual conclusion of genetic susceptibility’: ‘positive textual conclusion of genetic susceptibility’ (OGSF_0000031), ‘negative textual conclusion of genetic susceptibility’ (OGSF_0000032) and ‘neutral textual conclusion of genetic susceptibility’ (OGSF_0000033). Using the vaccine adverse event example, a ‘positive textual conclusion of genetic susceptibility’ means that a positive conclusion is drawn based on a significant statistical association of a genetic factor and a vaccine adverse event as identified in a published paper. A ‘negative textual conclusion of genetic susceptibility’ denies such a possible association between a genetic factor and an adverse event as declared in a published paper. Sometimes, depending on the data, an investigator might not be able to draw a definitive positive or negative conclusion on a genetic susceptibility association. This situation is captured using ‘neutral textual conclusion of genetic susceptibility’. In addition, OGSF also provides several datatype properties, such as ‘hasOddsRatio’ and ‘hasPvalue’, to allow the representation of digital data for statistical evaluation of the textual conclusion of genetic susceptibility (Figure 2).

### Use case studies

Case studies are used for two purposes: 1) to validate the modeling, 2) to test possible applications of the ontology. Below we represent two case studies reported from peer-reviewed journal articles using the OGSF framework.

#### Case study 1: HLA allele DBR1*15:01 is genetic susceptibility to Pandemrix related multiple sclerosis in a case report study

Pandemrix is an influenza pandemics vaccine that is developed by the company GlaxoSmithKline. The vaccine Pandemrix is represented in the Vaccine Ontology (VO) with the VO ID: VO_0000410. Vrethem *et al.* reported the occurrence of severe Multiple Sclerosis (MS) in a previously healthy young male in association with the vaccination of Pandemrix [34]. In this study, a human DBR1*15:01 allele is responsible for association with the Pandemrix-related MS adverse event. DBR1*15:01 is an allele of human leukocyte antigen (HLA) complex that encodes a MHC class II cell surface receptor. The association of this allele with MS appears to be consistent with many previous reports on situations other than vaccine adverse event [35,36].

**Figure 3:**
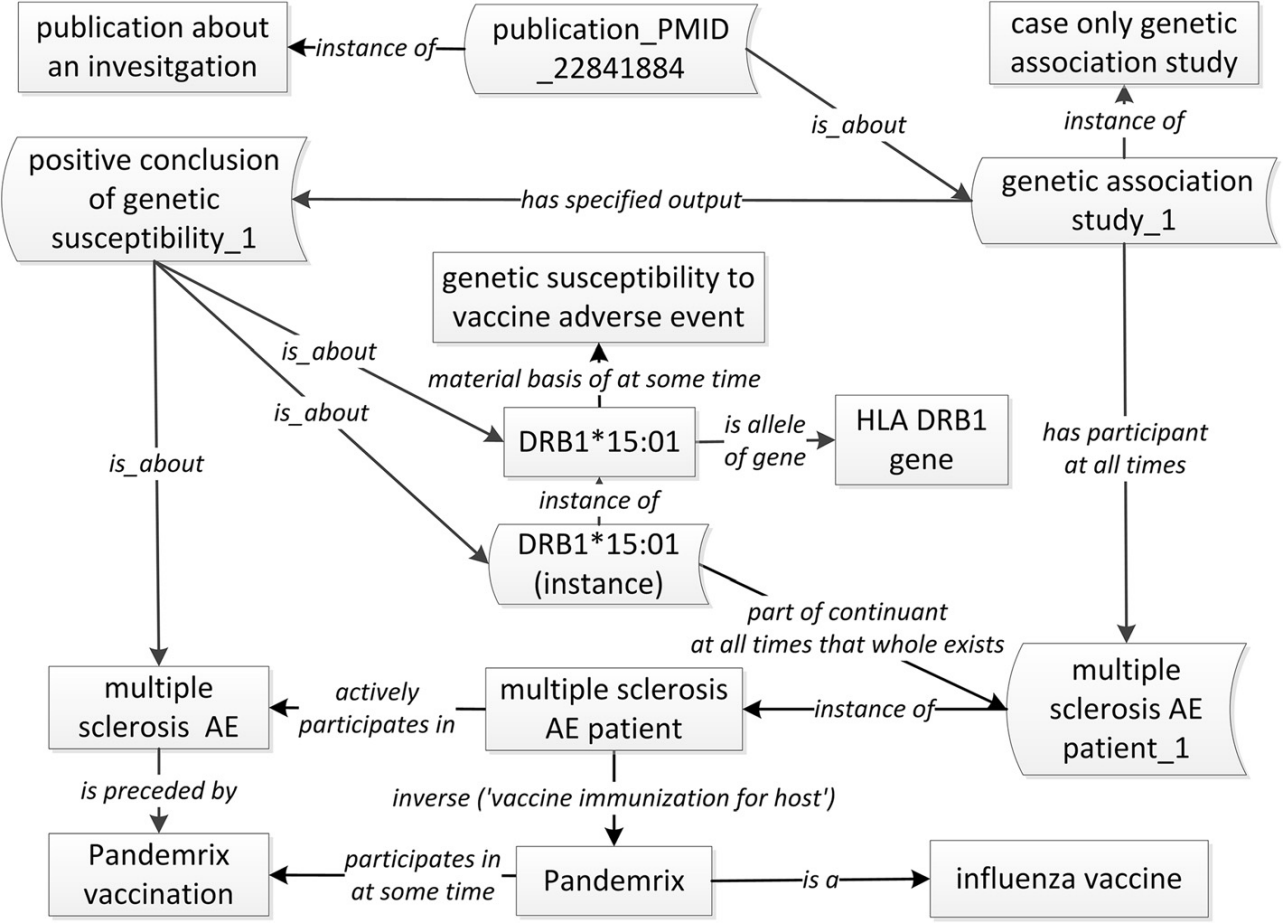
**OGSF modeling of vaccine-associated multiple sclerosis.** Square boxes denote classes, and curved box denote instances.

This genetic susceptibility case was represented in Figure 3 by following the general OGSF design pattern (Figure 2). For ontological modeling, it is critical to generate description logic constraints and axioms to accurately represent human- and computer-interpretable knowledge. As an example, the basic information about DRB1*15:01 can be ontologically represented as:

• ‘DRB1*15:01’ is subclass of ‘allele of gene’.

• ‘DRB1*15:01’ is subclass of (is_allele_of_gene some ‘HLA DBR1 gene’).

In addition to the above basic logical definitions, genetic susceptibility related to ‘DBR1*15:01’ can be identified based on different studies. Case Study 1 is such a study, which is represented as ‘genetic association study_1’ (Figure 3). This study generated a specific output ‘positive conclusion of genetic susceptibility_1’. This specific conclusion ‘is about’ the class ‘DBR1*15:01’ and the ‘multiple sclerosis AE’. The instance of ‘DBR1*15:01’ is a part of the specific patient in the case study. Based on this and many other case reports [34-36], we have generated the OGSF representation at the class level:

• ‘DRB1*15:01’ is subclass of (‘part of continuant at all times that whole exists’ some (‘human vaccinee’ and (inverse (‘vaccine immunization for host’) some Pandemrix)))

• ‘DRB1*15:01’ is subclass of (‘material basis of at some time’ some ‘genetic susceptibility to vaccine adverse event’)

• ‘DRB1*15:01’ is subclass of ‘susceptibility allele’

This case study indicates that OGSF provides necessary elements to represent genetic susceptibility and genetic susceptibility factors associated with vaccine adverse events.

#### *Case study 2: genetic polymorphisms associated with adverse events after smallpox vaccination in multiple clinical trials*

Reif *et al.* reported that genetic polymorphisms in several genes encoding important immune factors, including enzyme methylenetetrahydrofolate reductase (MTHFR), an immunological transcription factor (IRF1), and interleukin-4 (IL-4), were associated with adverse events after smallpox vaccination [37]. In this report, two independent clinical trials were conducted as initial and replicating genetic association studies. Different from the Case Study 1 where an allele of gene is a susceptibility factor, susceptibility alleles of Single Nucleotide polymorphisms (SNPs) are the material basis of genetic susceptibility in this Case Study 2. Table 1 lists all the SNPs (*e.g.*, the A allele of rs839 SNP in the gene *irf1*), their associated genes, and the Odds Ratio and p-value from two clinical trials [37].

**Table 1:** Statistical summary of genetic susceptibility factors with systemic adverse event following smallpox vaccination

**GSF**^**&**^	**Allele**	**Gene**	**Odds ratio (confidential interval)**	**P-value**	**Study 1 or 2**
rs1801133 SNP	T	MTHFR	2.3 (1.1–5.2)	0.04	1
rs1801133 SNP	T	MTHFR	4.1 (1.4–11.4)	0.01	2
rs9282763 SNP	G	IRF1	3.2 (1.1–9.8)	0.03	1
rs9282763 SNP	G	IRF1	3.0 (1.1–8.3)	0.03	2
rs839 SNP	A	IRF1	3.2 (1.1–9.8)	0.03	1
rs839 SNP	A	IRF1	3.0 (1.1–8.3)	0.03	2
Haplotype 1*	G,A	IRF1	3.2 (1.0–10.2)	0.03	1
Haplotype 1*	G,A	IRF1	3.0 (1.0–9.0)	0.03	2
Haplotype 2^#^	T,C,A	IL4	2.4 (1.0–5.7)	0.05	1
Haplotype 2^#^	T,C,A	IL4	3.8 (1.0–14.4)	0.06^$^	2

Notes:

^&^GSF stands for Genetic Susceptibility Factor.

*Haplotype 1 contains G allele of rs9282763, and A allele of rs839 in IRF1 gene.

^#^Haplotype 2 contains T allele of rs2070874, C allele of rs2243268, and A allele of rs2243290 in IL4 gene.

^$^In the second study, Haplotype 2 didn’t achieve the significant level of p value.

The data of this table is summarized and curated from Reif. *et al.*’s work on the genetic analysis of adverse event after smallpox vaccination [37].

**Figure 4:**
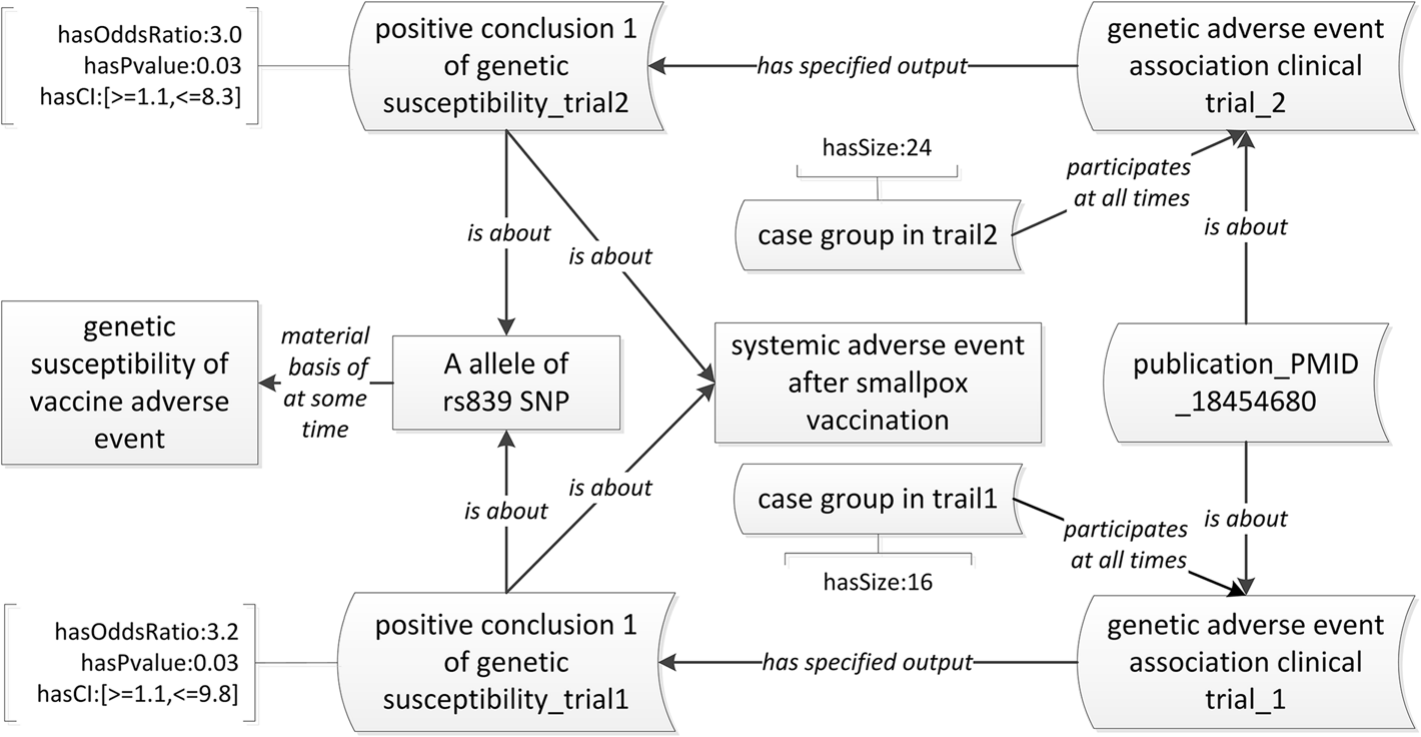
**OGSF modeling of case study 2.** Square boxes denote classes, and curved boxes denote instances.

The OGSF design pattern was applied to represent the information from these clinical trial studies (Figure 4). This figure does not include many linkages and axioms similar to those illustrated in Figure 3. Instead, Figure 4 focuses on representation of statistics providing evidence indicating the type of genetic associations to vaccine adverse events. In OGSF, the datatype property ‘hasSize’ allows the recording of the size of a human study subject group such as ‘case group’. The datatype properties ‘hasOddsRatio’, ‘hasPvalue’ and ‘hasCI’ (confidence interval) link the corresponding data to specific textual conclusion of genetic susceptibility. The Odds Ratio, P-value, and confidential interval are used to measure the association between genotypes and vaccine adverse event [37]. The Odds Ratio represents the ratio that an outcome will occur given an exposure, compared to the odds of the outcome occurring in the absence of the same exposure [38]. Using these datatype properties, the values of these measurements were captured and represented within the ontology. For example, the conclusion of clinical trial 1 regarding the ‘T allele of rs1801133 SNP’ was supported by the statistical data: having an Odds Ratio of 2.3, a P-value 0.03, and a confidence interval of [> = 1.4, <=11.4]. These statistical results support a positive genetic association between the allele of SNP and systemic adverse events of smallpox vaccination [37].

Since OGSF provides a framework to ontologically represent the complex data structure (including different variables and relations among these variables), the representation of the knowledge and data using OGSF supports computer-assisted data integration and reasoning. Such data sets can be queried efficiently using SPARQL as described below.

#### *SPARQL query*

The SPARQL Protocol and RDF Query Language (SPARQL) is the query language and protocol for the Resource Description Framework (RDF) data. RDF decomposes any knowledge into triples. Each RDF triple contains three components: subject, predicate, and object [39]. OGSF is developed using the Web Ontology Language (OWL) [40]. Both RDF and OWL are means to express increasingly complex information or knowledge, and both can be serialized in the RDF/XML syntax. RDF by itself has a limited capability for formal knowledge representation. OWL adds ontological capability to RDF by defining the components of RDF triples with formal computable first order description logic. So OWL provides more “semantic richness”. In addition, the OGSF OWL document can be converted to RDF format and queried by SPARQL.

From the OGSF supported knowledge system, our questions are focused on: 1) the list of susceptibility factors to a certain disease or pathological bodily process; 2) the evidences, either supportive or negative, supporting those susceptibilities. Using Case Study 2 as an example, we designed a SPARQL query to identify the genetic susceptibility factors to systemic adverse event of smallpox vaccination and related statistical evidences. The SPARQL script developed to query against the OGSF ontology is provided as follows:

This query was executed in the SPARQL plugin embedded with Protégé 4.3, build 304, and it could also be performed using the SPARQL endpoint (http://www.ontobee.org/sparql/index.php) in Ontobee [41], a linked data webserver where OGSF was deployed. The SPARQL execution retrieved five susceptibility factors to systemic smallpox vaccine adverse event as shown in Additional file 1 and listed below:

1. ‘T allele of rs1801133 SNP’ supported by 1 positive evidence.

2. ‘G allele of rs9282763 SNP’ supported by 2 positive evidence.

3. ‘A allele of rs839 SNP’ supported by 2 positive evidence.

4. ‘haplotype 1 in IRF1 gene’ supported by 2 positive evidence.

5. ‘haplotype 2 in IL4 gene’ supported by 1 positive evidence, and 1 negative evidence.

The SPARQL query output is consistent with the results obtained from the paper (Table 1). Therefore, our evaluation confirms the value of OGSF ontology representation of genetic susceptibility knowledge and instance data set.

#### *Social network analysis and visualization*

After an ontology is generated, it is often valuable but challenging to determine which ontology terms are more central and carry more information than other terms in the ontology. As an ontology defines terms and relations (object properties) between terms, an ontology can be viewed a social network. Specifically, the terms and relations of an ontology can be viewed as a directed hyperlinked graph G = (V, E) with nodes v∈V and edges e∈E, where the nodes correspond to the terms or entities in an ontology, and a directed edge (p, q) ∈ E indicates the relation that links from *p* (*i.e.*, the relation’s domain) to *q* (*i.e.*, the relation’s range). Therefore, the methods used for social network analyses may be potentially used for identifying key ontology terms as hubs or clusters of ontology terms [42]. In this study, we aimed to apply known social network analysis methods to evaluate the structure of the OGSF ontology and examine whether OGSF was constructed effectively to represent key entities for study of genetic susceptibility and genetic susceptibility factors as we designed.

Social Network Analysis (SNA) is the sum of the tools and methodologies of graph theory to analyze and thus describe structures of social networks [43]. Many SNA methods also overlap with network analysis methods from other domains such as literature mining-derived gene network analyses [44]. Two questions have been pre-designed for such social network analyses: Firstly, can the use case data support such identified central terms in the network? Secondly, can different network analysis methods generate different results and insights? To address these questions, the data from Case Study 2 were extracted using OntoGraf [45], and then visualized and analyzed using social network visualization tool Gephi [46]. The software was used to conduct the analyses of the degree centrality, closeness centrality, and hubs and authority scores to measure the relative importance of a node within the network. The statistical measurement data of these analyses are included in Additional file 2.

**Figure 5:**
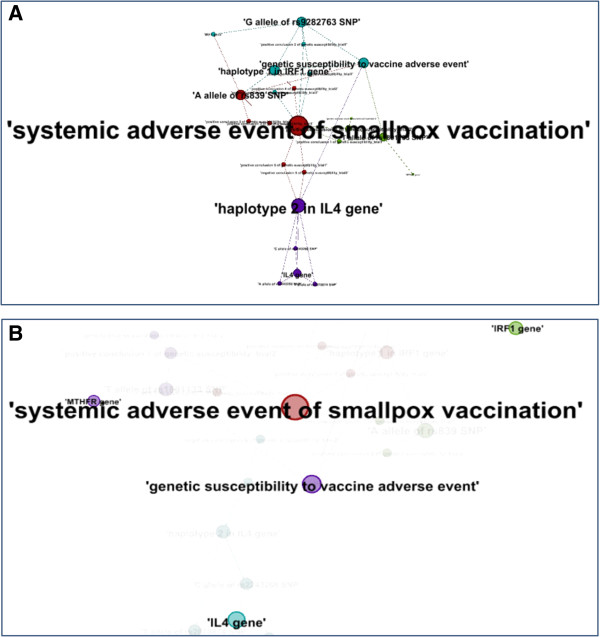
**Degree and closeness network analyses using Case Study 2 data modeled in OGSF. (A)** Degree centrality. The size of a node indicates the degree of the node indicating the number of connections from the node. **(B)** Closeness centrality. The closeness centrality analysis identified all three genes in the case study dataset. The visible nodes in the figure all have closeness centrality value equal to ‘0’. The nodes in the figure represent classes and instances contained in the case study. Those nodes displayed in the same color are clustered in the same group by the modularization method of the software Gephi [46].

The first method of our network analysis was based on the calculation of the degree centrality (Figure 5A). The degree centrality is simply the number of direct edges that an entity has in a network [43,44]. The network has 24 nodes and 38 edges with an average degree of 1.538. Our analysis found that the two terms with the highest degree centrality scores are ‘systemic adverse event of smallpox vaccination’, and ‘haplotype 2 in IL4 gene’. These two terms have the highest numbers of links to other terms. These findings are consistent with the knowledge stored in the ontology. However, the term ‘haplotype 2 in IL4 gene’ is not our intended core terms. This gives us insights that the degree measurement only cannot verify the core terms of the current network.

Secondly, we used the closeness centrality for network exploration (Figure 5B). The closeness centrality measures the average shortest path from a node to all other nodes. Specifically, the closeness centrality calculates the inverse of the farness that is the sum of a node’s distances to all other nodes [47]. The more closeness centrality a node is, the easier it can be reached by other nodes or reach out other nodes. The five ontology terms that have the best closeness centrality scores and have no out-reaching nodes are ‘genetic susceptibility to vaccine adverse event’, ‘systemic adverse event following smallpox vaccination’, ‘IL4 gene’, ‘IRF1 gene’, and ‘MTHFR gene’. The result is consistent with the design and construction of the ontology: the evidence link to ‘*genetic susceptibility*’ and ‘*vaccine adverse event’*, the variants link to *‘genes’*. It is interesting that all the three genes were identified together in this study.

**Figure 6:**
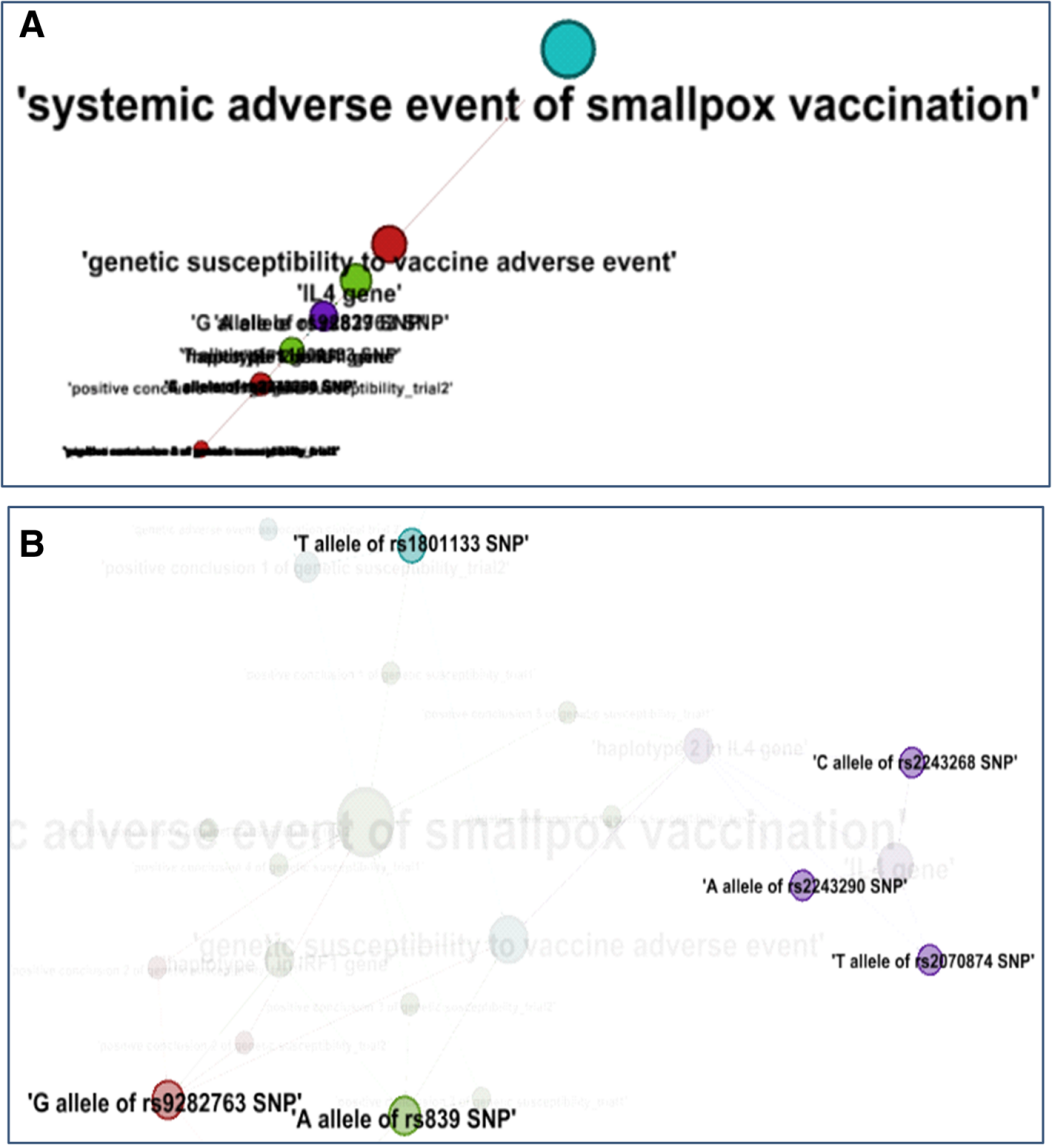
**Authority and hub network analyses using Case Study 2 data modeled in OGSF. (A)** Authority analysis. The top 3 node with the highest authority score are ‘systemic adverse event of smallpox vaccination’, ‘genetic susceptibility to vaccine adverse event’, and ‘IL4 gene’. **(B)** Hub analysis. Hub nodes in this network are all the SNPs. All the visible nodes have the highest hub score of 0.08.

The third network analysis was based on the calculation of the authority and hub scores [47,48] (Figure 6). The terms (nodes) that many other terms point to are called authorities. In contrast, the terms pointing to a relatively high number of authorities are called hubs. The authorities and hubs are a natural generalization of the eigenvector centrality that measures the influence of a node in a network. The authority analysis has been used for ranking web pages, and the data and ontologies from the Semantic Web search [49]. Figure 6A shows that top three authority centralized nodes: ‘systemic adverse event of smallpox vaccination’, ‘genetic susceptibility of vaccine adverse event’, and ‘IL4 gene’. There results indicate: 1) the main focus of this piece of linked data is about systemic adverse event of smallpox vaccination and genetic susceptibility; 2) IL4 gene carries more information flow than others, for it is connected with two kinds (positive and negative) of evidence and a haplotype of three SNPs in the network. Figure 6B shows nodes with highest hub scores. Interestingly, these identified hubs are all the SNPs related to the adverse event concluded in Case Study 2.

In summary, different network characteristics calculations reflect different dimensions of the ontology knowledge. The closeness and authority centrality analyses verified the core terms of the OGSF dataset in case study 2 are ‘systemic adverse event of smallpox vaccination’ and ‘genetic susceptibility of vaccine adverse event’. Interestingly, the hub analysis identified all the alleles of SNPs, and the closeness analysis detected all three hidden genes that are related to those alleles of SNPs. It is noted that the genes instead of the alleles of SNPs are usually found by direct literature searching. Based on these observations, our network analyses accurately identified ontology terms essential for representing genetic susceptibility and genetic susceptibility factors.

## Discussion

In this paper, we have introduced the development of the new version of the Ontology of Genetic Susceptibility Factors (OGSF) and its usage for ontologically representing genetic susceptibility to vaccine adverse events. The new OGSF is aligned with the BFO 2.0. OGSF imports many terms from existing ontologies and also includes many new ontology terms. For the first time, we have ontologically represented the genetic susceptibility to a pathological bodily process (*i.e.*, vaccine adverse event). Two vaccine adverse event use cases were represented and evaluated. The SPARQL and social network analyses were implemented to evaluate and analyze the OGSF contents and structure. Different social network analysis methods identified ontology terms with different types of importance in the ontology.

OGSF emphasizes the classification of different genetic factors and polymorphisms associated with susceptibility to diseases or pathological bodily processes. Some susceptibility factors may be genotype or mutation, which can be expressed using different allele classes. Moreover, OGSF has several classes, such as susceptibility SNP interval, susceptibility gene, and susceptibility haplotype to host those entities that is not allele per se. For example, in the constructed network of our case study 2, the ‘IL4 gene’ is the third authoritative node but the first gene identified from the authority analysis (Figure 6A). From the SPARQL query result, only ‘haplotype 2 of IL4 gene’ is linked to two different evidences: the positive conclusion from trial 1 and the negative conclusion from trial 2. Moreover, the ‘haplotype 2 of IL4 gene’ is consisted of three SNPs that is more than other haplotype in the network (Table 1). This structure increases the ranking of IL4 gene in the authority analysis comparing to other genes. More interestingly, in another genetic susceptibility to smallpox vaccine adverse event study, a haplotype in IL4 gene is related with a decrease of the susceptibility to fever after vaccination [50]. This haplotype contains a SNP rs2243250 located in the promoter region of IL 4 gene, where a C → T substitution is associated with increased production of IL-4 [50]. Searching the HaploReg database [51], this SNP is predicted to be located in the same haplotype of IL4 gene introduced in Case Study 2. This example shows the complicated role that IL4 gene polymorphisms play in the systemic adverse event triggered by smallpox vaccination. It also shows the importance of representing the increase or decrease (resistance) of genetic susceptibility.

In addition to the genetic susceptibility factors, many other variables may also contribute to the manifestation of a disease or a pathological bodily process outcome (*e.g.*, vaccine adverse event) [30]. For example, the human individual’s characteristics, such as race/ethnic identity, geographical region, and disease history, may also play an important role in the manifestation of an adverse outcome. Different genetic study design, such as family study or population-based study, may lead to different conclusions. To identify possible causality between a genetic susceptibility factor and a VAE, a statistical analysis is often required. The sample size of human subjects involved will also affect the statistical power of genetic association studies. Our integrative OGSF framework has incorporated many statistical terms in order to measure the robustness of the genetic association with a specific disease or pathological outcome. The statistical measurement then gives foundations to support the true genetic association between genetic susceptibility factors and related disease or pathological bodily process. Well-designed experiments may be applied to verify the association.

Different methods can be used for ontology evaluations [52]. A use case analysis is critical to evaluate the correctness, completeness, and utility of an ontology. Two use cases have been chosen and presented in the paper to illustrate how OGSF is logically constructed and useful in representing genetic susceptibility to vaccine adverse events. To further evaluate the ontology utility in addressing specific questions, we designed and implemented SPARQL queries to identify known genetic susceptibility factors to smallpox vaccine-induced systemic adverse events as shown in the second use case. Furthermore, different social network analyses were applied to identify and verify the key ontology terms essential in the topic.

Although social network analysis (SNA) has been widely used in the fields of web search and social studies, its application in ontology field is rare. SNA uses graph theories. Since ontologies can be considered as (labeled, directed) graphs, graph analysis techniques are promising tools for evaluating ontologies in many dimensions. Hoser *et al.* have applied SNA to analyze the structures of Suggested Upper Merged Ontology (SUMO) and SWRC ontology [43]. Harth *et al.* and Hogan *et al.* have been developing search strategies using network-based approaches to mine linked data in semantic web respectively [49,53]. Their studies show that the SNA of a given ontology provides deep insights into the structure of ontologies and knowledge base. These ontology-related SNA studies treated all ontology classes and relations as network nodes. Different from this approach, our SNA analyses only consider ontology classes and their instances as nodes and make ontology relations (*i.e.*, object properties) as edges. Our distinct treatment of ontology relations as edges makes senses since these relations are designed to link different classes and their instances. Our SNA study found that the visualization and social network analysis results using the Case Study 2 data provide better understanding of ontology designing and evaluation. Interestingly, our SNA hub and closeness analyses generated two distinct sets of results. The hub analysis identified all five susceptibility alleles of SNPs as top key terms while the closeness analysis detected all three susceptibility genes collected in the Case Study 2. The SNA hubs are terms directed to the high authority terms. Our identification of all the SNPs as hubs is consistent with the notion that these SNPs are essential for the authority terms such as ‘systemic adverse event of smallpox vaccination’ and ‘genetic susceptibility of vaccine adverse event’. The closeness centrality measures how a node can be easily reached by other nodes. As the genes have different susceptibility variants (*i.e.*, SNPs of genes), it makes sense that the genes have better closeness centrality scores than their variants. Since these genes are not directly defined as genetic susceptibility factors, the genes appear to be hidden factors that can be mined from the OGSF data. When we consider the gene functions, the direct gene name extraction gives more biological meaningful information than the variants themselves. These distinct observations suggest that different SNA analysis methods may identify ontology terms essential from different aspects.

Other than OGSF, many other research projects also focus on establishing and cataloging the relation between genotypes and phenotypes. For example, the Database of Genotypes and Phenotypes (dbGaP) is a repository for archiving, curating, and distributing the information obtained from studies investigating the interactions of genotypes and phenotypes [54]. SNPedia is focused on the medical, phenotypic and genealogical associations of SNPs [55]. The Leiden Open (source) Variation Database (LOVD) provides open data of genetic variants curated from published paper, and the disease association information is included [56]. GWAS central (previously called HGBASE, HGVbase and HGVbaseG2P) provides a centralized compilation of summarized findings from genetic association studies [57]. These resources provide structured raw or curated information related to genotypes and phenotypes. However, unlike OGSF, these resources do not ontologically represent different genetic susceptibility types and genetic susceptibility factors with all necessary information and evidence assertions. OGSF is able to serve as an intermediate and an integrative layer between various evidence-based medicine applications and above existing structure data resources and other unstructured data resources.

Our study clearly shows that OGSF provides a robust platform to support logical representation and analysis of genetic susceptibility and genetic susceptibility factors. Such platform will allow us to logically organize the knowledge and data related to genetic susceptibility and genetic susceptibility factors. With the well-organized information, it is then possible to generate automatic reasoning programs to analyze the data, predict new knowledge on genetic susceptibility, and support personalized medicine research. However, while the use case studies out of the literature curation were meant for evaluating and validating the OGSF framework, it would be a huge effort to manually curate all the possible data available in the literature. To improve the study of genetic susceptibility factors, it might help to devote more programing effort to selectively integrate related data sources from openly accessible resources such as the SNPedia [55] as introduced above. Advanced text mining programs may also be developed to retrieved related information from unstructured literature data. Following these programming efforts, a large amount of manual curation may also be requested for expanding the ontology and making it more useful. To achieve a long-term goal of solving susceptibility issues, some specific domains may initially be focused. We are looking for collaborations for further applying OGSF for practical usage for scientific domains.

## Conclusions

Originated from previous OGSF-DM research [14], the new Ontology of Genetic Susceptibility Factors (OGSF) is aligned with the framework of BFO 2.0 and developed to ontologically represents various genetic susceptibility types, genetic susceptibility factors, and related entities and relations. OGSF has been used to represent genetic susceptibility and susceptibility factors associated with vaccine adverse events as annotated from experimental studies. Our SPARQL and network evaluations have shown that OGSF is able to provide a robust framework for the representation and analysis of genetic susceptibility knowledge and datasets. The social network analysis results also demonstrated that key ontology terms critical in different aspects can be detected with different centrality-based network analysis methods.

## Methods

### Ontology editing

The format of OGSF ontology is W3C standard Web Ontology Language (OWL2) (http://www.w3.org/TR/owl-guide/). For this study, many new terms and logical definition were added into original OGSF [14] using the Protégé 4.3.0 build 304 OWL ontology editor (http://protege.stanford.edu/).

### Ontology term reuse and new term generation

OGSF imports the whole set of the Basic Formal Ontology (BFO) [58]. To support ontology interoperability, terms from OBO Foundry ontologies, such as OBI, OAE, IAO and etc., are reused. For this purpose, OntoFox [59] was applied for extracting individual terms from external ontologies. For those genetic susceptibility-specific terms, we generated new OGSF IDs with the prefix of “OGSF_” followed by seven-digit auto-incremental digital numbers. New OGSF terms created according to the intensive modeling from the use cases.

### Evaluation of OGSF by SPARQL

Use case studies were designed based on literature survey. SPARQL was performed using the SPARQL query plug-in embedded with Protégé 4.3.0 build 304.

### Evaluation of OGSF by social network analysis

Graphed data used for visualization was first extracted from OGSF using the OntoGraf plug-in [44]. After manual editing, the file (Additional file 3) was used as input for the network visualization software Gephi 0.8.2 beta (http://gephi.org) [45]. Gephi was also used to conduct social network data analysis and visualization based on the extracted data. The embedded algorithms in Gephi were used to calculate the scores of degree, closeness [59], and hub and authority [46].

### Availability and access

The website for OGSF project is available at http://code.google.com/p/ogsf/. As an OBO Foundry library ontology, OGSF has been deposited by default in the Ontobee linked data server [41]. All OGSF terms can be browsed and searched via the Ontobee at http://www.ontobee.org/browser/index.php?o=OGSF. The source of the ontology is also deposited in the NCBO Bioportal: http://bioportal.bioontology.org/ontologies/3214.

## Abbreviations

BFO: Basic formal ontology; FOAF: Friend of a friend project; HLA: Human leukocyte antigen; GAZ: Gazetteer; IAO: Information artifact ontology; LD: Linkage disequilibrium; OAE: Ontology of adverse event; OBI: Ontology for biomedical investigations; OBO: Open biological and biomedical ontologies; OGDI: Ontology of genetic disease investigation; OGI: Ontology for genetic interval; OGMD: Ontology of glucose metabolism disorders; OGMS: of General medical science; OGR: Ontology of geographical regions; OGSF: Ontology of genetic susceptibility factors; OGSF-DM: Ontology of genetic susceptibility factors to diabetes mellitus; OVAE: Ontology of vaccine adverse event; OWL: Web ontology language; REO: Reagent ontology; SKOS: Simple knowledge organization system; SNA: Social network analysis; SNP: Single polymorphism nucleotide; SPARQL: SPARQL protocol and RDF query language; SUMO: Suggested upper merged ontology; URI: Uniform resource identifier; VO: Vaccine ontology.

## Competing interests

The authors declare that they have no competing interests.

## Authors’ contributions

YL: Primary OGSF developer, use case testing, social network analysis and visualizing, drafting of manuscript. YH: OGSF developer, vaccine adverse event domain expert, use case testing, and drafting of manuscript. Both authors read and approved the final manuscript.

## Supplementary Material

Additional file 1 **Screen shots of SPARQL queries. (A)** SPARQL query used in Ontobee SPARQL query endpoint. The file includes the SPARQL query script used in the Ontobee SPARQL query endpoint (http://www.ontobee.org/sparql/index.php) and its results as returned by the Ontobee SPARQL query server. **(B)** Additional File 4.png. Screen shot of Protégé SPARQL query tab showing the SPARQL query result. Click here for file

Additional file 2 **Network characteristic measurements of each node in the use case 2 graph.** The file includes in-degree, out-degree, degree, authority, hub, modularity, clustering, strength, local clustering coefficient, eigenvector centrality, PageRank, eccentricity, closeness centrality, betweenness centrality scores of the 24 nodes in the graph. The calculation was conducted by using Gephi software. Click here for file

Additional file 3 **The input file used for network visualization analysis using Gephi software.** The file includes the data of individuals and related classes of case study 2 extracted from OGSF ontology. Click here for file
