# Supplementary material for: The ontology of genetic susceptibility factors (OGSF) and its application in modeling genetic susceptibility to vaccine adverse events
Source: J Biomed Semantics. 2014 Apr 30;5:19. doi: 10.1186/2041-1480-5-19 (PMC4068904; doi:10.1186/2041-1480-5-19)
Supplement: Additional file 1 — Screen shots of SPARQL queries. (A) SPARQL query used in Ontobee SPARQL query endpoint. The file includes the SPARQL query script used in the Ontobee SPARQL query endpoint (http://www.ontobee.org/sparql/index.php) and its results as returned by the Ontobee SPARQL query server. (B) Additional File 4.png. Screen shot of Protégé SPARQL query tab showing the SPARQL query result. [file 2041-1480-5-19-S1.pdf]

SPARQL query result using the SPARQL plugin embedded with Protégé 4.3, build 304:

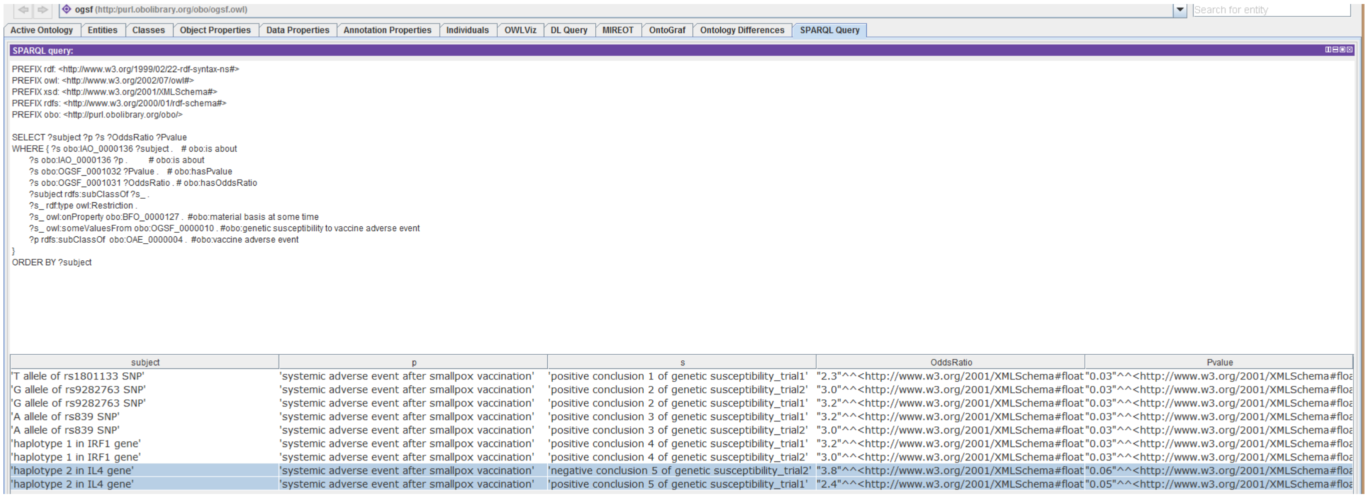

The SPARQL query code and returned result when using Ontobee SPARQL query endpoint (http://www.ontobee.org/sparql/index.php)

SPARQL Query code:

```
# query using ontobee SPARQL query endpoint:
PREFIX obo: <http://purl.obolibrary.org/obo/>
SELECT ?subject_label ?p_label ?s_label ?OddsRatio ?Pvalue ?CI
from <http://purl.obolibrary.org/obo/merged/OGSF>
WHERE { ?s obo:IAO_0000136 ?subject .
        ?s obo:IAO_0000136 ?p .
        ?s rdf:type obo:OGSF_0000031 .
        ?s obo:OGSF_0001032 ?Pvalue .
        ?s obo:OGSF_0001031 ?OddsRatio .
        ?s obo:OGSF_0000040 ?CI .
        ?subject rdfs:subClassOf ?s_ .
        ?s_ rdf:type owl:Restriction .
        ?s_ owl:onProperty obo:BFO_0000127 .
        ?s_ owl:someValuesFrom obo:OGSF_0000010 .
            ?p rdfs:subClassOf  obo:OAE_0000004 .
        ?subject rdfs:label ?subject_label .
        ?s rdfs:label ?s_label .
        ?p rdfs:label ?p_label .
```

Ontobee returned result :

Result | Raw Request/Permalinks | Raw Response

subject_label	p_label	s_label	OddsRatio	Pvalue	CI
G allele of rs9282763 SNP	systemic adverse event after smallpox vaccination	positive conclusion 2 of genetic susceptibility_trial1	3.200000047683716	0.02999999932944775	[>=1.1, <=9.8]
G allele of rs9282763 SNP	systemic adverse event after smallpox vaccination	positive conclusion 2 of genetic susceptibility_trial2	3	0.02999999932944775	[>=1.1, <=8.3]
A allele of rs839 SNP	systemic adverse event after smallpox vaccination	positive conclusion 3 of genetic susceptibility_trial1	3.200000047683716	0.02999999932944775	[>=1.1, <=9.8]
haplotype 1 in IRF1 gene	systemic adverse event after smallpox vaccination	positive conclusion 4 of genetic susceptibility_trial2	3	0.02999999932944775	[>=1.0, <=9.0]
haplotype 2 in IL4 gene	systemic adverse event after smallpox vaccination	positive conclusion 5 of genetic susceptibility_trial1	2.400000095367432	0.05000000074505806	[>=1.0, <=5.7]
T allele of rs1801133 SNP	systemic adverse event after smallpox vaccination	positive conclusion 1 of genetic susceptibility_trial1	2.299999952316284	0.02999999932944775	[>=1.4, <=11.4]
A allele of rs839 SNP	systemic adverse event after smallpox vaccination	positive conclusion 3 of genetic susceptibility_trial2	3	0.02999999932944775	[>=1.1, <=8.3]
haplotype 1 in IRF1 gene	systemic adverse event after smallpox vaccination	positive conclusion 4 of genetic susceptibility_trial1	3.200000047683716	0.02999999932944775	[>=1.0f, <=10.2f]
